# Remote Guidance of Untrained Turtles by Controlling Voluntary Instinct Behavior

**DOI:** 10.1371/journal.pone.0061798

**Published:** 2013-04-17

**Authors:** Serin Lee, Cheol-Hu Kim, Dae-Gun Kim, Han-Guen Kim, Phill-Seung Lee, Hyun Myung

**Affiliations:** 1 Department of Civil and Environmental Engineering, Korea Advanced Institute of Science and Technology, Yuseong-gu, Daejeon, Republic of Korea; 2 Division of Ocean Systems Engineering, Korea Advanced Institute of Science and Technology, Yuseong-gu, Daejeon, Republic of Korea; University of Cambridge, United Kingdom

## Abstract

Recently, several studies have been carried out on the direct control of behavior in insects and other lower animals in order to apply these behaviors to the performance of specialized tasks in an attempt to find more efficient means of carrying out these tasks than artificial intelligence agents. While most of the current methods cause involuntary behavior in animals by electronically stimulating the corresponding brain area or muscle, we show that, in turtles, it is also possible to control certain types of behavior, such as movement trajectory, by evoking an appropriate voluntary instinctive behavior. We have found that causing a particular behavior, such as obstacle avoidance, by providing a specific visual stimulus results in effective control of the turtle's movement. We propose that this principle may be adapted and expanded into a general framework to control any animal behavior as an alternative to robotic probes.

## Introduction

Several artificial intelligent agents, such as micro- and nano-aerial vehicles (MAVs/NAVs), have been developed for the performance of tasks which humans cannot easily handle. However, these agents have not performed as well as expected due to the limitations of size/weight, battery capability/charging, range of operation, and so on. A major lesson, thus far, is that we are still far from artificially reproducing a level of intelligence even of insects. Thus, interest in alternative approaches based on biologically inspired or biomimetic methods has increased.

Recent work on the direct control of lower animal behavior has focused on the measurement of operating range and speed, versus payload and maneuverability, and on studies of animal social behavior [1]. Several mechanical control systems have been reported, such as the insect flight control system proposed by Sato et al., which electronically stimulates the insect's brain and muscles in charge of its flight [2]; the wireless communication device of Britt et al., which provides commands to a well-trained dog [3]; the remote flight control system of Tsang et al., which uses micro-fabricated flexible neuroprosthetic probes integrated with carbon nanotube-gold nano-composites in a moth [4]; and the 2.5-mW wireless insect flight controller designed by Daly et al., which utilizes a non-coherent pulsed ultra-wideband receiver system-on-chip (3–5 GHz) [5]. In studies of differential brain stimulation, Talwar et al. have shown that rats are easily guided by specific stimulation of either the somatosensory cortical (SI) or medial forebrain bundle (MFB) as a cue or reward, respectively [6]. Most proposed behavior control systems require a well-trained animal or cause involuntary behavior by direct stimulation of the corresponding musculature by an implanted controller. In insects, implantation is carried out at the adult or pupal stage.

Our study has addressed the problem of control in two fundamentally different aspects: whether we can control an untrained animal in a non-invasive and remote manner, and if this may be done via control of voluntary behavior. Our results indicate this is indeed the case. All animals, including humans, usually act by reaction to stimuli. In particular, a reactive behavior connected with bodily protection is essential and must occur quickly, and it must be evoked, mediated, and directed in a consistent manner by a stimulus [7], [8]. From these studies in turtles, we have observed a consistent pattern of control of an animal's movement trajectory utilizing the innate instinctive behavior of obstacle avoidance, and we propose this as a novel behavior control scheme. Using this non-invasive scheme, our system of animal behavior control can be more stable and adoptable. The system is suitable for application in tasks traditionally carried out by mobile robots, such as surveillance and reconnaissance, exploration and navigation, as well as other missions dangerous for humans.

We first conducted experiments to investigate in detail the turtle's obstacle avoidance behavior, in which we took advantage of earlier work on the turtle's vision wavelength discrimination [9] and the observation that hatchling sea turtles recognize a white light source as an open space and so move toward it [10], [11].

## Materials and Methods

### Turtles

**Figure 1:**
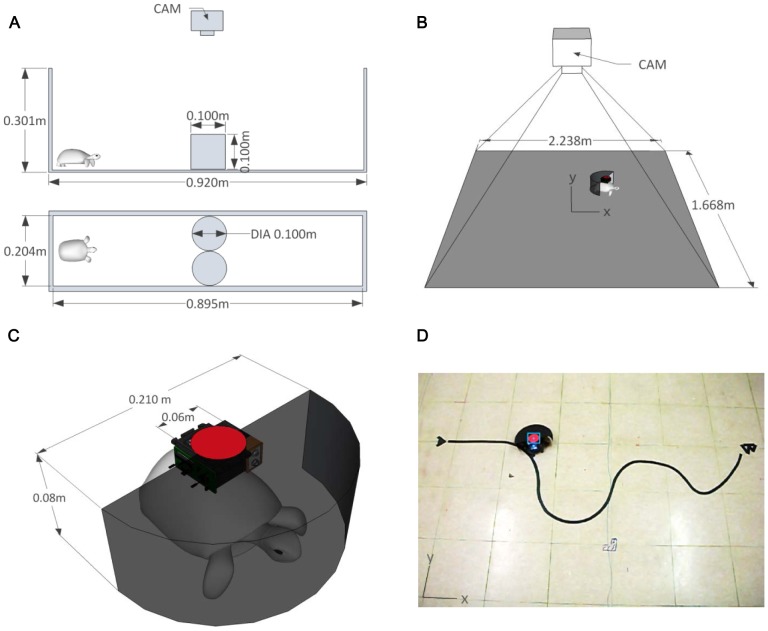
Depiction of experimental remote-controlled visual stimulus delivery and tracking systems. (A) To examine the turtle's visual obstacle recognition, an experimental arena was equipped with a camera and two movable cylinders as obstacles (shown from the side view and from above). The dimensions of the arena, surrounding walls, and obstacles are indicated. (B) Experiments performed on the laboratory floor area (with the dimensions indicated) are shown in the drawing. The placements of the turtle, obstacle, and tracking system are shown. (C) The embedded control system to block the turtle's view is shown in the drawing. The servo motor controls the positioning of the semi-cylinder obstacle (in the image, it is positioned directly in front of the turtle). The red circle on the controller tracked by the simple tracking algorithm was regarded as the location of the turtle. (D) The turtle was remotely controlled to follow the desired path by alternating the visual angle of the obstacle between ±180 (no stimulus) and ±90 degrees (Movie S1).

We decided to do our experiment with turtles because it is easy to detect their movement, and they are capable of living in various types of habitats on land and in water. The turtles used in this study were red-eared sliders (*Trachemys scripta elegans*). Four turtles were grown indoors in laboratories at the Korea Advanced Institute of Science and Technology (KAIST). The turtles were housed together in a large, water-filled glass tub (91×61×20 cm). The tank was fitted with a water filter and a dry platform for basking, and the turtles were sunbathed 6∼7 hours under a UV lamp. They were fed commercial pellets four times a week. After at least 6 hours without feeding in the tank, they were moved to the floor of the laboratory or the experimental table for experiments as shown in Figure 1. As each experiment was repeated, the turtles became sluggish from fatigue; therefore, different turtles were used for our experiments every 10 minutes. Thus, we carried out the experiments using all four turtles (Figure 1).

### Method

**Figure 2:**
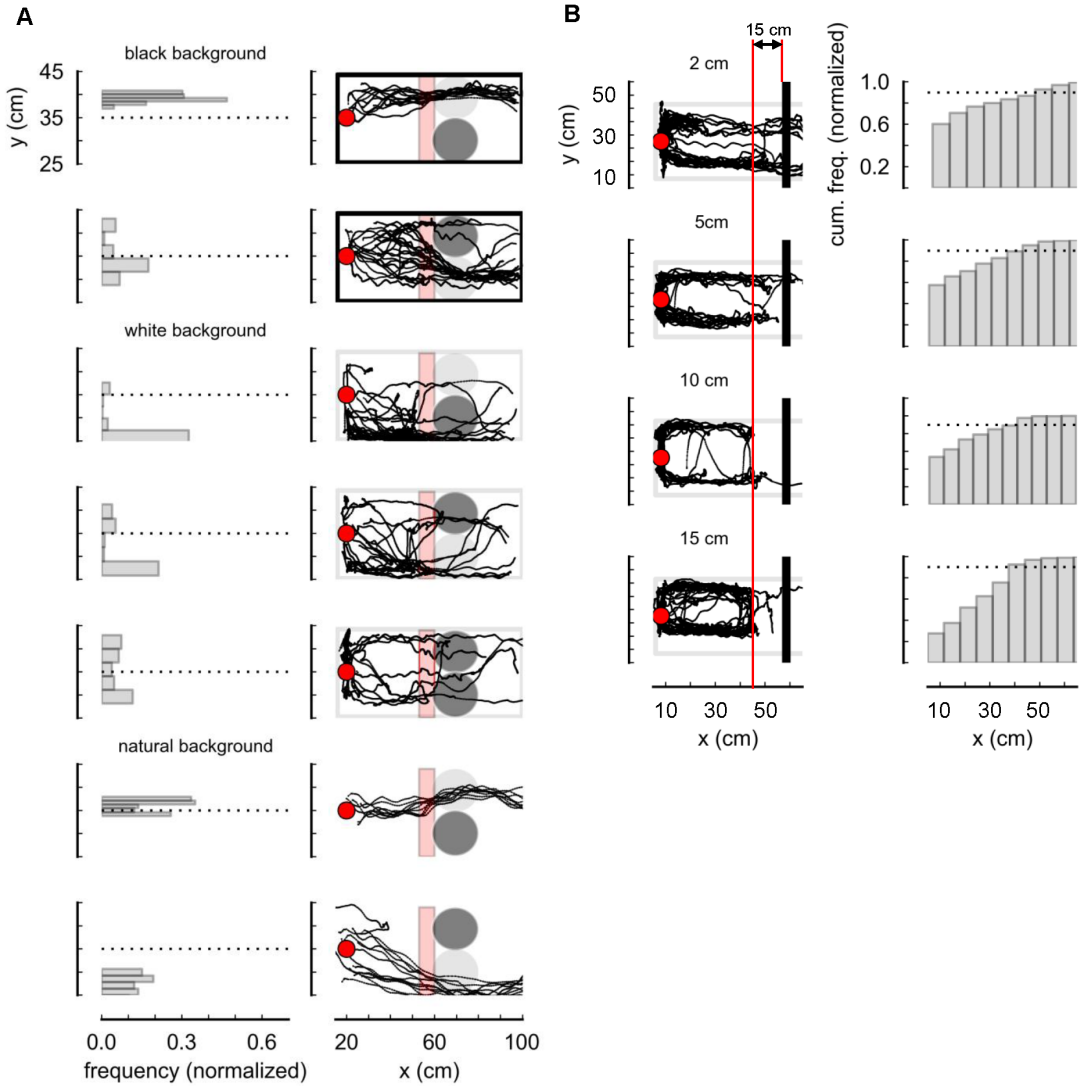
Control of obstacle avoidance behavior. (A) The movement trajectories were tracked after turtles (red circles) were initially placed 50 cm in front of obstacles. Obstacles were movable black or white cylinders (radius = 5 cm, height = 10 cm), and the turtles could push them to go past. (B) Turtles were initially located 55 cm (marked by red circles at mid-carapace) in front of a movable black obstacle wall in an arena with white side walls.

**Figure 3:**
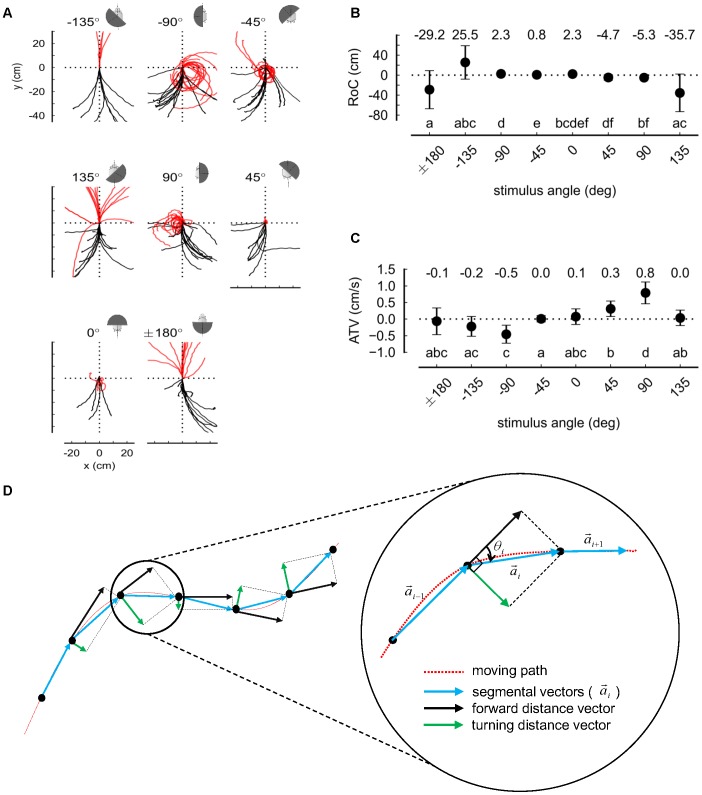
Relationship between a turtle's movements and visual angle to the obstacle. (A) Each movement trajectory was translated to place the location at which the stimulus was given at the origin, and then rotated so that its tangential line at that location coincides with the y-axis. The red lines represent the trajectories after the visual stimuli were provided. The black lines describe the trajectories before the stimuli. The angle of the obstacle is indicated numerically and by the image of the semi-cylinder. Two black dotted lines show orientation and comparison (n = 10–21). (B) The radii of curvature (RoC) of the red trajectories in (A) are plotted by mean and standard deviation, although they were not always normally distributed. (C) The average turning velocities (ATV) of the trajectories are plotted and analyzed as described for the RoC in (B). (D) To measure the turning behavior, a turning distance vector was defined as shown in this figure (see text for details).

As mentioned in the Introduction, this study aimed to control turtle's behavior by providing visual stimuli. We therefore examined how turtles respond to various visual stimuli. The experiments were performed in arenas on the experiment table (90×20 cm) (Figure 2) and the floor (223.8×166 cm) (Figure 3). The turtles' responses, that is, their navigational paths, were continuously recorded by a simple color-based tracker. Except for our target stimulation, other factors (olfactory stimuli, auditory stimuli, room temperature, brightness distribution, etc.) were controlled during the experiments.

Each turtle's path was tracked by a 20 Hz digital camera (VLUU NV4, SAMSUNG, KOREA) with 800×592 pixel resolution. The center of a circular color patch (radius = 3 cm) attached to the center of the turtle's upper shell was tracked by a color-based tracker that used a MATLAB (The Mathworks Inc., USA) image processing program developed by Maptic (See http://www.matpic.com). The average distance between the center of the patch and the end of the turtle's head was 6 cm.

The raw data from the color-based tracker was post-processed by projective transformation mapping of the oblique view to the top view, and a Kalman filter with linear models was used for both the dynamics of the system and the observation process. Like other Bayesian-based tracking algorithms [12], the parameters of the filter were carefully chosen by an iterated trial-and-error procedure comparing the filtered and real trajectories by eye, and we found that we could obtain good results under the covariances of *Q* = 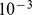 and *R* = 0.1. The whole system is described in Figure 1.

### Apparatus

To provide the turtle with stimulus causing obstacle avoidance, a simple control device was designed to locate a semi-cylinder at any given angle with respect to the anteroposterior axis of the turtle. An embedded control module (5.3×7.5×4.8 cm, 133.5 g) was mounted on the turtle's upper shell with the circular color patch for tracking, and a black semi-cylinder was used to block the turtle's view. A micro controller unit (ARM Cortex-M3, STM32F101V8T6) received an angular value to control the servo motor (Maximum output angle: 2,160 degrees, Resolution: 4.9 degrees, Motorbank, KOREA), which could rotate the black semi-cylinder within ±180 degrees with respect to its body axis, from a PC control software written in C# via Bluetooth communication (Baud rate: 19,200 bps, Firmtech, KOREA).

### Ethical Note

The *Trachemys scripta elegans* were manipulated under the following animal permits from the Korea Advanced Institute of Science and Technology according to the KAIST Animal Experiment Ethical Law RR0303, last changed on 10/6/2009. Our animal experiment qualification certifications are Cheol-Hu Kim (2010-OE01), Serin Lee (2011-CE01) and Dae-Gun Kim (2011-OE01).

## Results

### Visual Recognition of Obstacles

There have been several studies on turtle's vision [9], but no research has addressed what kinds of objects the turtles recognize as obstacles. Therefore, by examining the turtle's movement trajectories when a black and a white cylinder (radius = 5 cm, height = 10 cm) were initially placed 30 cm in front of it, we found that the turtle recognized the black cylinder as an obstacle, rather than the white cylinder. Figure 2A shows the experimental results. The color of the wall surrounding the arena varied: black, white, or a natural scene. In Figure 2A, avoidance tendency, described by a turtle's location when it was less than 7.5 cm from an obstacle (pink-shaded region), is shown in histograms. The turtles were placed in an arena with black side walls facing toward a white obstacle, or with white side walls. With white side walls (recognized as an open space), most turtles moved through the narrow passageway between a wall and the cylinder (n = 14–27).

### Obstacle Recognition Distance

To examine the obstacle recognition distance, a black wall was placed 55 cm in front of a turtle in an arena with 10 cm-high white side walls. Black walls of various heights (2, 5, 10, and 15 cm) were used to test if obstacle recognition distance also depends on the obstacle's apparent size. The turtle's walking trajectories were recorded by the color-based tracker as described in Figure 1. The experimental results are shown in Figure 2B. In this figure, the histograms indicate the approach distance to the obstacle wall as cumulative frequency. Trajectories were aimed in the direction of white side walls. In more than 90% of the trials (dotted line in the histogram), the turtles did not come closer than 15 cm from the obstacle, regardless of its height (apparent size) (n = 10–25).

### Visually Planned Obstacle Avoidance

To test how turtles respond to obstacles in more detail, we provided stimulus by utilizing a device mounted on the turtle's carapace (Figure 1D and Movie S1). Since the turtles recognized the black object closer than 15 cm as an obstacle as shown in the previous experiments, a simple device, which could stimulate obstacle avoidance behavior, was designed to locate a black semi-cylinder (radius = 10.5 cm, height = 8 cm) at any specific angle in front of it. Since this can give a constant stimulus to the turtle, this could be considered as a device for an open-loop experiment that can amplify behavioral responses by removing a turtle's visual feedback [13].

When the black semi-cylinder only blocked the turtle's view horizontally, no meaningful results were obtained. We then found it necessary to simultaneously block horizontal and vertical views by placing a top cover on the device. We also believe that the controlling factor is the location of the black/white edge relative to the front of the eye, but for convenience, we used the center of the circumference of the semi-cylinder instead. The location of the center of its circumference can be thus varied from +180 to −180 degrees in a clockwise direction with respect to the anteroposterior axis of the turtle. Since a turtle shows little response to a light emitted from ±180 degrees [10], we assumed that a semi-cylinder located at ±180 degrees could be regarded as providing no stimulus. After the turtle walked for 5.0 sec with no stimulus, the black semi-cylinder was positioned in front of it at a specific angle within ±180 degrees and kept in this position for 30 sec (Figure 3A). We then tracked the turtle's walking path under this condition. Throughout this experiment, the turtles continued to move around in a circle when they were exposed to constant visual stimuli, until they got tired.

For such experiments, we introduced the concept of average turning velocity (ATV) to measure the amount of displacement or shift from the turtle's previous heading per unit of time. After the turning distance (TD) is derived by dividing the entire path into n-segmental vectors by sampling each second, the ATV under the condition θ≤90 degrees can be defined by
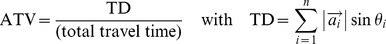
as shown in Figure 3D. In this experiment, the more a turtle's view was blocked by an obstacle, the sharper it turned away from the stimulus (Figure 3A). The radius of curvature (RoC) is then described as shown in Figure 3B. In this figure, the two-tailed Mann-Whitney U test (p<0.05) and Bonferroni correction was performed for statistical comparisons of the data sets. The results shown in lower case are shown as statistically homogeneous groups [14]. For example, the groups ‘a’ and ‘b’ are significantly different; but ‘a’ is not significantly different from ‘ab’, which shares membership with group ‘a’. Although the conventional curvature only considers the change of angle, the ATV takes both the change of angle and distance traveled into consideration. In the case of ±135 degrees, the ATV was small since the change of angle was small; on the other hand, the ATV for ±45 degrees was small because the distance traveled was short. The maximum absolute ATV values were obtained when the black semi-cylinder was located at ±90 degrees (Figure 3C).

### Controlling Turtle's Walking Path

**Figure 4:**
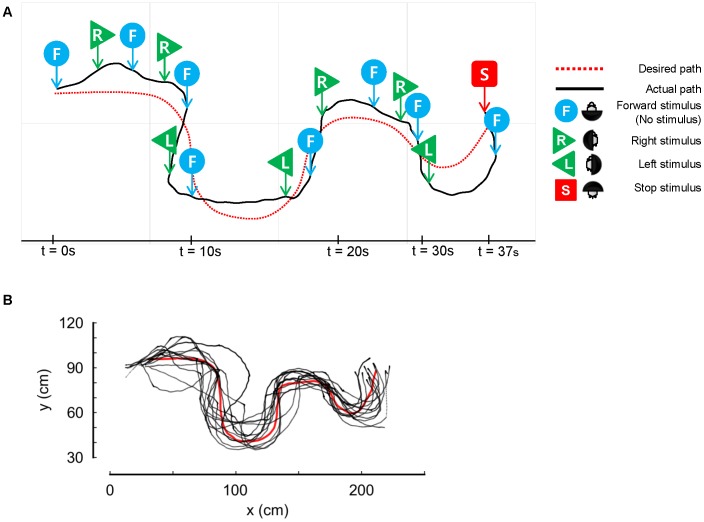
Controlling turtle's walking path. (A) Examples of guided turtle navigation using the embedded control system to block the turtle's view. Each arrow indicates positions at which forward (F), stop (S), right (R) and left (L) directional stimuli were issued. (B) Turtle movement was controlled by alternately providing forward, right, left and stop stimuli causing obstacle avoidance (see text). The desired (red) and actual (black) paths of the turtles are plotted.

In most trials, when a turtle's view was largely blocked, it became immobile. On the other hand, a small degree of blockage did not affect a turtle's path. We therefore attempted to remotely cause a change in the path of a moving turtle by alternating between no stimulus (±180 degrees) and that of ±90 degrees (Figure 4 and Movie S1). During these experiments, we also discovered that immobile turtles (occasionally induced by a moderate stimulus) could be prompted to begin moving again by remotely waving the cover of the obstacle.

## Discussion

In this study, we examined one of the essential responses for an organism's survival: obstacle avoidance. By providing a visual stimulus that causes the behavior, we remotely controlled a turtle's walking behavior. We first examined the turtle's visual recognition of obstacles under various conditions. We found that the turtles, recognizing the white object as open space, headed for it regardless of other conditions. Second, we tried to find out the turtle's obstacle recognition distance. We discovered that no matter what the obstacle's height (apparent size), the turtle did not come closer to it than 15 cm. Third, we then designed a simple device to examine the turtle's visually planned obstacle avoidance behavior. We found that the more the turtle's view was blocked by the obstacle, the sharper it turned away from it. Lastly, by applying the above results, we were able to successfully control the turtle's walking paths.

These experiments demonstrate that animal behavior can effectively be guided by evoking instinctive behavior essential for survival. Unlike the involuntary behavior control schemes that have been previously proposed, which compel a response by stimulating the corresponding neural circuit (or musculature) regardless of the animal's intention, our approach is to guide the animal by elaborately inducing its voluntary instinctive behavior. In addition, while most involuntary controllers may require additional sensors to adjust responses to an abrupt or unexpected situation (e.g., when an insect meets an uncontrolled obstacle in its otherwise controlled or planned path), voluntarily controlled animals are expected to adapt themselves to the situation by combining the directed and adaptive behaviors.

We therefore believe that an innate behavior caused by a visual stimulus can easily and effectively be employed to control an animal's movement, and will not impose a heavy strain on the animal. Although it is necessary to overcome the technical difficulties of designing a new device in order to apply the specific stimulus causing the innate behavior to other animals in other environments, this approach may provide a clue to a general framework for behavior control. In future works, we will study controlled behavior in more detail and also apply this framework to other animals that have excellent vision. Hawks, cats, lizards and carp are good candidates. They are also big and strong enough to carry larger devices. Through our on-going research, we already found that the same framework can be employed to control fish.

While in this study turtles were controlled in a well-prepared experimental setup, our final goal is to build a device to control animals in real environments. To achieve this goal, we will face a lot of challenges related to miniaturization, waterproofing, telecommunication, and navigation at the least. We expect the most useful behavior controller will incorporate nonlinear control methods with a positioning system (like indoor GPS) and IMU (Inertial Measurement Unit) to direct animal behavior without any human intervention. This technology could be used in deep sea exploration and could replace our dependence on robotic probes. We could also take such opportunities to observe their movement from an ethological perspective to understand their complicated social behavior.

## Supporting Information

Movie S1 **Control of movement trajectory in turtles by stimulation of obstacle avoidance behavior.**
(AVI)
